# Light-induced increase in the steady-state chlorophyll fluorescence in cyanobacteria reflects induction of energy dissipation complementary to orange carotenoid protein-dependent thermal dissipation

**DOI:** 10.1007/s11120-025-01159-0

**Published:** 2025-07-09

**Authors:** Takako Ogawa, Hiroko Takahashi, Yoshitaka Nishiyama, Yukako Hihara, Kintake Sonoike

**Affiliations:** 1 Graduate School of Science and Engineering, Saitama University, 255 Shimo-Okubo, Sakura-ku, Saitama, 338-8570 Japan; 2 Faculty of Education and Integrated Arts and Sciences, Waseda University, 2-2 Wakamatsu-cho, Shinjuku-ku, Tokyo, 162-8480 Japan

**Keywords:** Cyanobacteria, Photosynthesis, Orange carotenoid protein, Phycobilisome, Chlorophyll fluorescence, Energy dissipation

## Abstract

Under high-light conditions, the dissipation of excess energy as heat in the light-harvesting antenna is essential for photosynthetic organisms to protect the photosynthetic machinery. In the case of cyanobacteria, however, the induction of the thermal dissipation in the antennae is insufficient to dissipate all excess energy, which is manifested as the increase in the steady-state level of chlorophyll fluorescence (Fs) under high light. To elucidate the underlying cause of the incomplete dissipation of excess light in the antenna, we investigated the impact of depletion and overexpression of orange carotenoid protein (OCP), which is essential to induce thermal dissipation in the antenna, on photosynthesis in *Synechocystis* sp. PCC 6803. The suppression of the OCP-dependent thermal dissipation resulted in elevated Fs with a constant yield of photosynthesis, suggesting that the light-induced increase in Fs might function as an acclimation mechanism to high light, which compensated for the lower OCP-dependent thermal dissipation. By contrast, over-induction of the OCP-dependent thermal dissipation decreased not only Fs but also the yield of photosynthesis under high light, due to the reduced energy transfer from the antenna to photosystem II. These results indicate that the complete removal of excess energy via the OCP-dependent mechanism has a drawback in photosynthetic efficiency under high-light conditions, and the strategy independent of OCP is employed to cope with excess light without lowering the yield of photosynthesis in cyanobacteria.

**Supplementary Information:**

The online version contains supplementary material available at 10.1007/s11120-025-01159-0.

## Introduction

All organisms must ever confront environmental changes. Among many environmental factors, light plays a significant role in photosynthetic organisms. Light is not only essential for photosynthesis and growth but also becomes dangerous when its intensity increases, since excess light causes over-reduction of the photosynthetic electron transport chain and promotes the production of reactive oxygen species, which brings about damage to the photosynthetic machinery (Murata et al. 2012). To minimize such risk under high-light conditions, the regulatory mechanism to dissipate excess energy as heat in the light-harvesting antenna of photosystem II (PSII) is essential (Rochaix 2014; Goss and Lepetit 2015).

In land plants, the accumulation of zeaxanthin (Demmig-Adams 1990) and the function of the PsbS protein (Li et al. 2000) are responsible for the thermal dissipation in the light-harvesting complex II (LHCII), which is triggered by the decrease in lumenal pH through the photosynthetic electron transport (Ruban et al. 2012; Rochaix 2014). In cyanobacteria, soluble phycobilisome serves as a light-harvesting antenna instead of LHCII, and orange carotenoid protein (OCP) is responsible for the thermal dissipation (Wilson et al. 2006; Kirilovsky 2015; Muzzopappa and Kirilovsky 2020). OCP acts as a light sensor, and intense 400–550 nm light converts OCP into a metastable form that induces thermal dissipation in the phycobilisome (Wilson et al. 2008).

Such thermal dissipation in antenna systems is reflected as non-photochemical quenching, which can be monitored by the saturation pulse method with a pulse amplitude modulation (PAM) fluorometer (Lazár 2015). Induction of the thermal dissipation in the antenna, which depends on the zeaxanthin, PsbS or OCP, has been evaluated by monitoring values of chlorophyll fluorescence parameters such as NPQ and Φ_NPQ_, which represent the so-called non-photochemical quenching (Bilger and Björkman 1990; Hendrickson et al. 2004), in land plants (Niyogi et al. 1998; Barbato et al. 2020) as well as in cyanobacteria (Takahashi et al. 2019). Upon increase in the photon flux densities (PFD), the yield of regulatory non-photochemical quenching (Φ_NPQ_) increases by the induction of thermal dissipation, along with the decrease in the yield of electron transport through PSII (Φ_PSII_) upon closure of the reaction center. The remaining fraction of absorbed energy, which is neither used for photosynthesis nor dissipated via the regulatory non-photochemical quenching-related mechanism, is represented by Φ_f, D_, and the sum of Φ_PSII_, Φ_NPQ_, and Φ_f, D_ is always unity: Φ_PSII_ + Φ_NPQ_ + Φ_f, D_ = 1 (Hendrickson et al. 2004). Φ_f, D_ has been described as the yield of “non-regulated” or “constitutive” non-photochemical dissipated energy, but its actual origin is not properly understood. High Φ_f, D_ was considered to represent low photoprotective capacity under excess light conditions, mainly due to the closure of PSII reaction centers (Klughammer and Schreiber 2008; Endo et al. 2014). It is reported that the steady-state level of Φ_f, D_ is relatively constant irrespective of the PFD of actinic light, i.e., the decrease in Φ_PSII_ under high light is covered by the increase in Φ_NPQ_ (Fig. S1, left panel), in many land plant species such as *Arabidopsis thaliana* (Kono et al. 2014; Barbato et al. 2020), *Oryza sativa* L. (Ishida et al. 2011; Ikeuchi et al. 2014), and *Vitis vinifera* L. (Hendrickson et al. 2004). Accordingly, the steady-state level of chlorophyll fluorescence (Fs) under continuous illumination, which primarily reflects the steady-state level of Φ_f, D_ (Hendrickson et al. 2004), is almost constant regardless of actinic PFD in land plants (Shrestha et al. 2012; Torres et al. 2022). Thus, in these conditions, Φ_NPQ_ can sufficiently dissipate excess energy brought about by the closure of PSII at steady state under high light in land plants.

The situation is, however, totally different in the case of cyanobacteria (Fig. S1, right panel). We observed that the steady-state level of Φ_f, D_ increases along with the increase in actinic PFD in *Synechocystis* sp. PCC 6803 (hereafter referred to as *Synechocystis* 6803; Ogawa et al. 2013), *Anabaena* sp. PCC 7120, *Nostoc* sp. HK-01, *Arthrospira platensis*, *Acaryochloris marina*, and *Nostoc punctiforme* (Misumi et al. 2016). These observations indicate that the increase in excess energy resulting from the decrease in Φ_PSII_ under high-light conditions cannot be fully covered by the increase in Φ_NPQ_, and that the induction of regulatory non-photochemical quenching is limited in these organisms. The limitation of regulatory non-photochemical quenching in cyanobacteria is also reflected in lower NPQ, another parameter representing non-photochemical quenching, compared to land plants. In *Synechocystis* 6803, NPQ is around 0.5 or lower even under illumination with intense blue light that induces OCP-dependent thermal dissipation (Kusama et al. 2015; Ogawa and Sonoike 2016; Takahashi et al. 2019). This value of NPQ is smaller than that in *A. thaliana*, in which NPQ often exceeds 2 under high-light conditions (Niyogi et al. 2001; Long et al. 2008; Li et al. 2009). Furthermore, the NPQ of the cyanobacterial OCP-deficient strain is around 0.4, which is only slightly lower than the value of its parental wild-type strain (Kusama et al. 2015; Takahashi et al. 2019). Compared with the case of *A. thaliana*, in which NPQ is decreased approximately from 2.5 to 0.5 by the defect in the zeaxanthin accumulation or PsbS (Niyogi et al. 2001), the impact of the defect in the OCP-dependent thermal dissipation on the capacity of regulatory non-photochemical quenching seems to be significantly smaller in *Synechocystis* 6803.

These observations led to several questions. Why is the capacity of the OCP-dependent thermal dissipation limited? Does excess energy cause no photoinhibitory effects on photosynthetic machinery in cyanobacteria in the absence of sufficient thermal dissipation at the antenna level? In this study, we investigated the impact of induction or suppression of the OCP-dependent thermal dissipation on steady-state photosynthesis in *Synechocystis* 6803 to reveal the physiological consequences of the insufficient dissipation of excess light energy at the antenna level in cyanobacteria. We found that suppression of the OCP-dependent mechanism brought about the increase in Φ_f, D_ corresponding to the decrease in Φ_NPQ_, resulting in little influence on the yield of photosynthesis, i.e., Φ_PSII_. By contrast, over-induction of the OCP-dependent mechanism lowered the yield of photosynthesis as well as Φ_f, D_ under high-light conditions. These findings indicate that cyanobacteria employ a complementary mechanism to the OCP-dependent thermal dissipation to cope with excess light without lowering the yield of photosynthesis, reflected in the increase in Φ_f, D_ under high-light conditions.

## Materials and methods

### Strains and growth conditions

The wild-type strain (WT), OCP-overexpressing strain (ox-*ocp*), and OCP-deficient strain (Δ*ocp*) of *Synechocystis* 6803 were grown at 30^o^C in BG11 medium, buffered with 20 mM TES-KOH (pH 8.0) and bubbled with air for 24 h under continuous illumination using fluorescent lamps from a side. PFD of the growth light was 120–130 µmol m^− 2^ s^− 1^ when measured by a spherical micro-sensor (US-SQS/L, Walz) with a light meter (LI-250 A, LI-COR Biosciences) inside the growth bottles. We used ox-*ocp* and Δ*ocp*, which had been generated previously, as well as the background WT of these transformants. Δ*ocp* was constructed by insertional mutagenesis (Kusama et al. 2015), and ox-*ocp* was constructed by incorporating the *ocp* gene, together with the *psbAII* promoter, into the neutral site in the genome (Takahashi et al. 2019). Chloramphenicol at 25 µg ml^− 1^ or kanamycin at 20 µg ml^− 1^ was added to the culture medium for the growth of ox-*ocp* or Δ*ocp*, respectively.

### Pulse amplitude modulated chlorophyll fluorescence measurements

Chlorophyll fluorescence was measured using a PAM fluorometer (MULTI-COLOR-PAM, Walz). Cell suspensions were adjusted to 2 µg chlorophyll ml^− 1^, and were dark-acclimated for 15 min before the measurements. The chlorophyll concentration of the cell suspensions was determined as described in Grimme and Boardman (1972). Actinic light (LEDs peaking at 440 nm or 625 nm) was applied for 2 min to determine Fs, followed by a 0.5 s flash of saturating light to determine Fm’, the maximum fluorescence level of the light-acclimated cells. Typical traces of the time-dependent fluorescence level (Ft) for the measurements of Fs and Fm’ are shown in Fig. S2. Fm, the maximal fluorescence level of chlorophyll fluorescence under conditions where both photochemical quenching and non-photochemical quenching were suppressed, was measured under illumination with actinic light in the presence of 10 µM 3-(3,4-dichlorophenyl)-1,1-dimethylurea. Fo’, the minimum fluorescence level of the light-acclimated cells, was calculated as Fo’ = Fo/{(Fm-Fo)/Fm + Fo/Fm’} (Oxborough and Baker 1997). Fs, Fm’, Fm, and Fo’ were used for the calculation of parameters; Φ_f, D_ = Fs/Fm, Φ_NPQ_ = Fs/Fm’-Fs/Fm (Hendrickson et al. 2004), Φ_PSII_ = (Fm’-Fs)/Fm’ (Genty et al. 1989), Fv’/Fm’ = (Fm’-Fo’)/Fm’ and qP = (Fm’-Fs)/(Fm’- Fo’) (van Kooten and Snel 1990).

### Chlorophyll fluorescence emission spectra at room temperature

Chlorophyll fluorescence emission spectra were measured at room temperature with a fluorescence spectrometer (FP-8500, JASCO) as described in Ogawa et al. (2013). Cell suspensions were adjusted to a chlorophyll concentration at 2 µg ml^− 1^. 440 nm light was used for chlorophyll excitation and 625 nm light for phycocyanin excitation, with an excitation slit width of 10 nm.

### Determination of wavelength-dependent absorption cross-section of PSII

The wavelength-dependent absorption cross-section of PSII, Sigma (II)_λ_, was determined as described in Schreiber et al. (2012). Sigma (II)_λ_ was calculated as Sigma (II)_λ_ = 1/(*Tau* × *N*_A_ × PFD). Here, *Tau* is the time constant of light-driven Q_A_ reduction, while *N*_A_ is Avogadro’s constant (6.022 × 10^23^ mol^− 1^). *Tau* was determined by measuring fluorescence kinetics upon illumination with orange (625 nm) or blue (440 nm) actinic light in the presence of background far-red light (725 nm) by a PAM fluorometer (MULTI-COLOR-PAM, Walz) and fitting the fluorescence kinetics curve by the PamWin-3 software (Walz) program. Cell suspensions, adjusted to 0.5 µg chlorophyll ml^− 1^, were exposed to orange or blue actinic light with increasing PFD ranging from 220 to 2521 µmol m^− 2^ s^− 1^ or from 1016 to 4018 µmol m^− 2^ s^− 1^, respectively. Single-turnover pulse for 50 µs was applied 1 ms after the onset of actinic light of each PFD to determine the maximum fluorescence level (I_1_-level) that can be reached in the presence of an oxidized plastoquinone (PQ) pool. To minimize potential errors in the fitting process due to the difference in the rate of fluorescence induction, Sigma (II)_λ_ was calculated with *Tau* determined under 510 µmol m^− 2^ s^− 1^ in the case of orange actinic light and 3123 µmol m^− 2^ s^− 1^ in the case of blue actinic light, which gave a similar rate of fluorescence induction (Fig. S3).

## Results

**Fig. 1:**
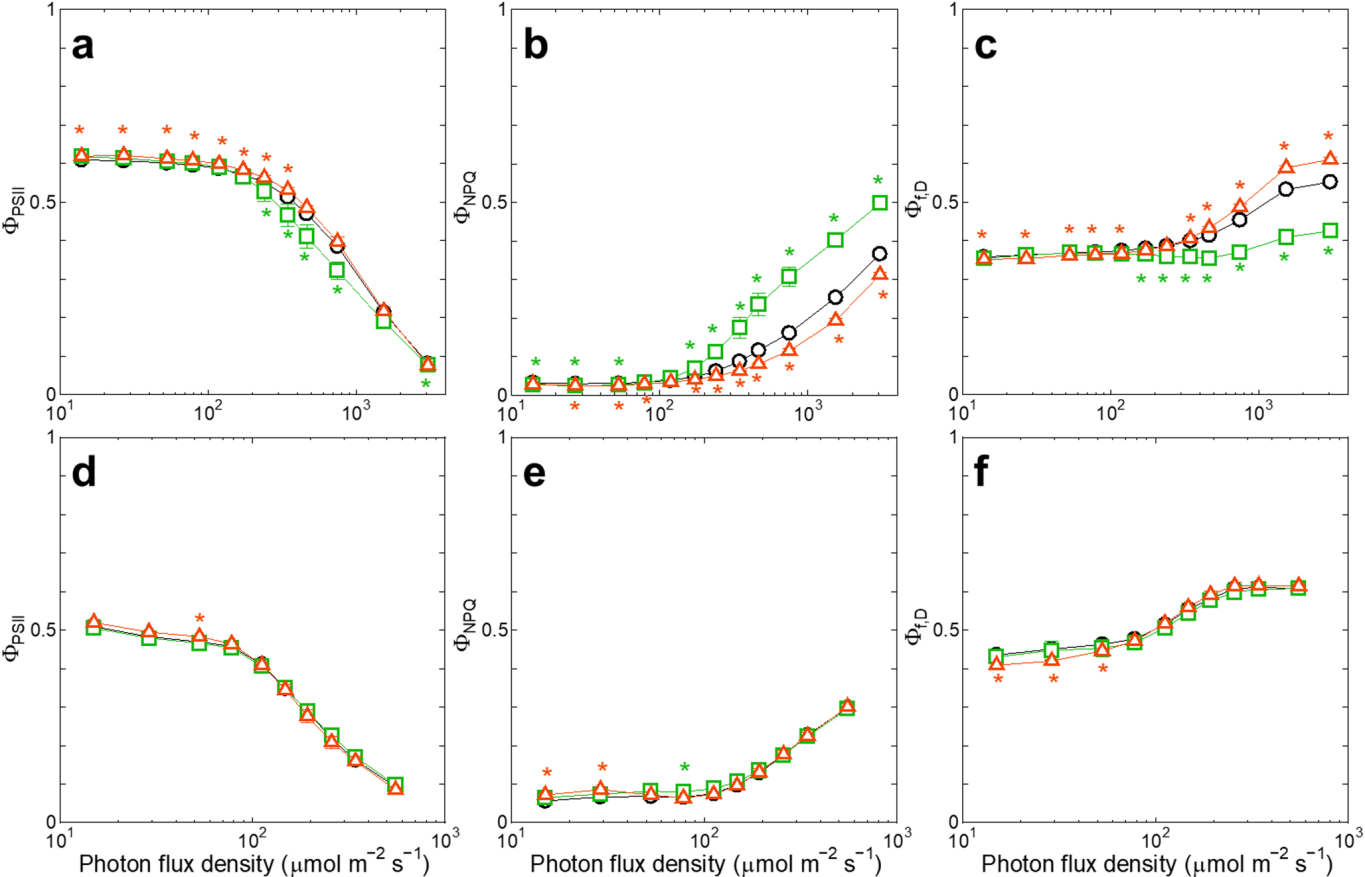
The response of Φ_PSII_, Φ_NPQ_, or Φ_f, D_ to PFD of blue (**a**-**c**) or orange (**d**-**f**) actinic light. The parameters are plotted on a logarithmic scale. Green squares, ox-*ocp*; orange triangles, Δ*ocp*; black circles, the background WT of the transformants. Averages ± SD of three independent cultures are presented. Green or orange asterisks indicate significant differences (*p* < 0.05) between WT and ox-*ocp* or Δ*ocp*, respectively

First, we examined the effect of blue actinic light (440 nm) on the energy partitioning among Φ_PSII_, Φ_NPQ_, and Φ_f, D_ (Fig. 1a-c), which were determined by the PAM chlorophyll fluorometer with blue measuring light. Upon exposure of *Synechocystis* 6803 cells to blue actinic light at PFD above 200 µmol m⁻² s⁻¹, a decrease in Φ_PSII_ was greater than an increase in Φ_NPQ_, resulting in an increase in Φ_f, D_ (Fig. 1a-c, black circles) as previously described. To ascertain the underlying cause of this insufficient regulatory non-photochemical quenching, we used the OCP-overexpressing strain (ox-*ocp*; Takahashi et al. 2019). The decrease in Φ_PSII_ in ox-*ocp* was comparable with the increase in Φ_NPQ_, resulting in smaller changes in Φ_f, D_ under high blue actinic light (Fig. 1a-c, green squares). The result suggests that the maximal potential of the OCP-dependent thermal dissipation is sufficient to cover the decrease in Φ_PSII_ under high light, if OCP is overexpressed. Conversely, the OCP-deficient strain (Δ*ocp*; Kusama et al. 2015) showed lower Φ_NPQ_ and higher Φ_f, D_ compared to WT, with a small effect on Φ_PSII_ (Fig. 1a-c, orange triangles), suggesting that the suppressed OCP-dependent mechanism was compensated by the induction of Φ_f, D_ under strong blue actinic light. These differences in Φ_PSII_, Φ_NPQ_ and Φ_f, D_ among the three strains were not observed under orange actinic light (625 nm) that does not induce the OCP-dependent thermal dissipation (Fig. 1d-f).

The steady-state level of Φ_f, D_ under constant actinic light is reflected in chlorophyll fluorescence measurements as Fs (Hendrickson et al. 2004). However, not only chlorophyll fluorescence but also phycobilisome fluorescence may contribute to the Fs signal in the PAM chlorophyll fluorescence measurements in cyanobacteria (Campbell et al. 1998; Ogawa and Sonoike 2016; Stirbet et al. 2019). To investigate whether the change in Fs was caused by a change in chlorophyll fluorescence of the PSII core complex or a change in phycobilisome fluorescence of the antenna, we compared the fluorescence emission spectra at room temperature between chlorophyll excitation (440 nm) and phycocyanin excitation (625 nm) (Fig. S4a). We observed two fluorescence emission peaks under phycocyanin excitation: one at around 660 nm due to phycocyanin and the other at around 685 nm due to chlorophyll *a* (red dashed line). Only the latter chlorophyll *a* peak was observed under chlorophyll excitation (blue solid line). These results suggest that the changes in Φ_f, D_ measured with blue measuring light (Fig. 1c, f) mainly reflected the changes in the fluorescence from chlorophylls, namely, from the PSII core complex rather than the phycobilisome antenna. This was also confirmed by comparing Fs with the chlorophyll-exciting and phycocyanin-exciting measuring light (Fig. S4b). Fs measured with the phycocyanin-exciting light (red closed circles) was higher than that measured with the chlorophyll-exciting light (blue open circles), reflecting a larger contribution from phycobilisome fluorescence, but the ratio of the increase in Fs by the high excitation PFD above 200 µmol m⁻² s⁻¹ is larger in the case of chlorophyll excitation. Thus, the increase in the steady-state level of Φ_f, D_ as well as that in Fs under high light (Fig. 1c, f) should reflect fluorescence emission at the PSII core level rather than at the phycobilisome antenna level.

**Fig. 2:**
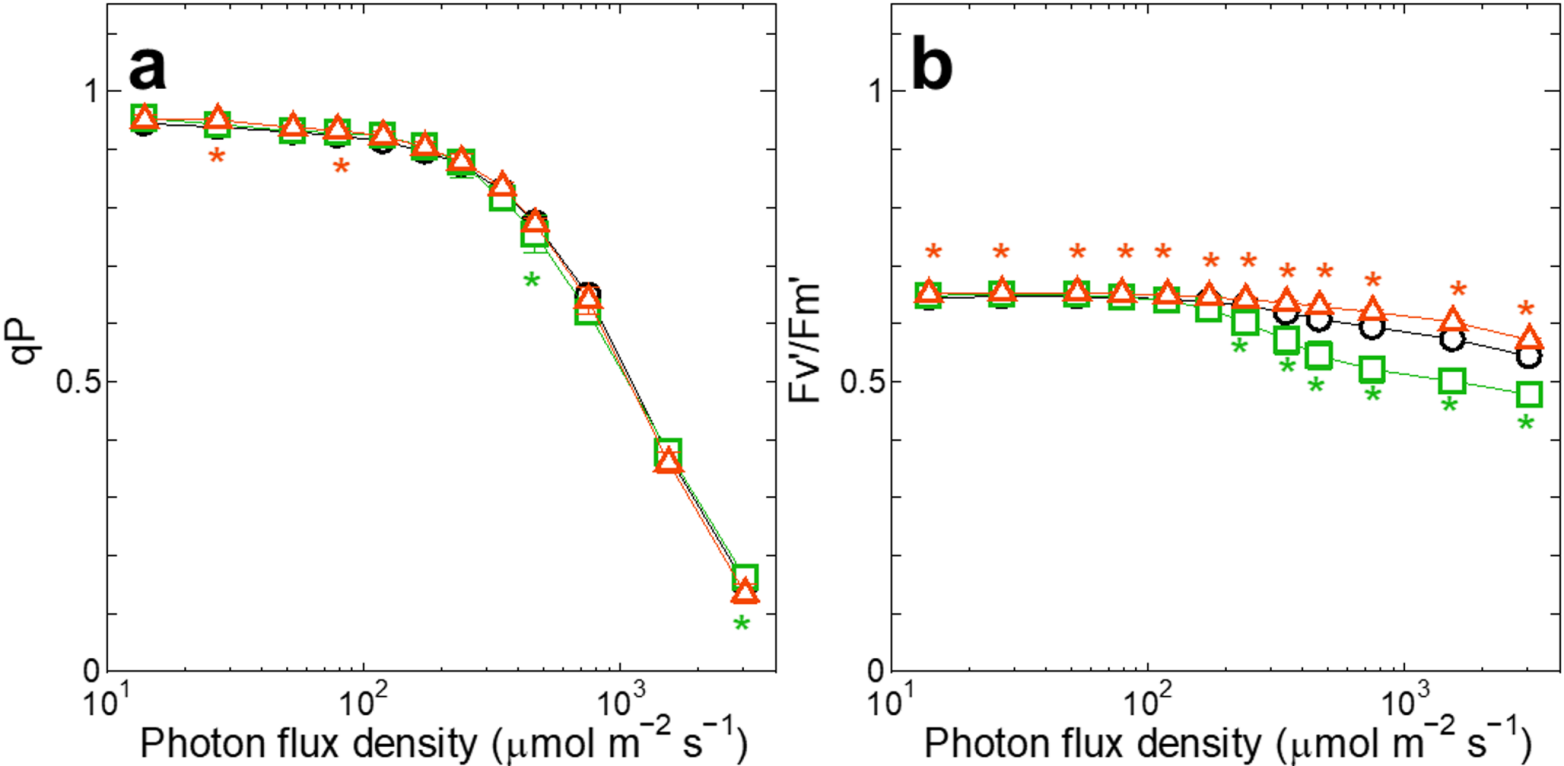
The response of qP (**a**) or Fv’/Fm’ (**b**) to PFD of blue actinic light. The parameters are plotted on a logarithmic scale. Green squares, ox-*ocp*; orange triangles, Δ*ocp*; black circles, the background WT of the transformants. Averages ± SD of three independent cultures are presented. Green or orange asterisks indicate significant differences (*p* < 0.05) between WT and ox-*ocp* or Δ*ocp*, respectively

To investigate whether the increase in Φ_f, D_ under strong blue light was caused by the closure of PSII, we compared the fraction of the open PSII (qP) among ox-*ocp*, Δ*ocp* and WT. Since there was no difference in qP among ox-*ocp*, Δ*ocp* and WT (Fig. 2a), we could not attribute the difference in the Φ_f, D_ level among the three strains (Fig. 1c) to the different redox state of Q_A_. Apparently, the induction of the OCP-dependent thermal dissipation was not so effective in avoiding the over-reduction of the photosynthetic electron transport chain even under strong blue light (Fig. 2a), but it decreased the efficiency of excitation capture by open PSII reaction centers (Fv’/Fm’) (Fig. 2b). Thus, as shown in Fig. 1a, it is only natural that Φ_PSII_ was smaller in ox-*ocp* than in WT under strong blue light, since Φ_PSII_ is the product of Fv’/Fm’ and qP (Genty et al. 1989).

**Fig. 3:**
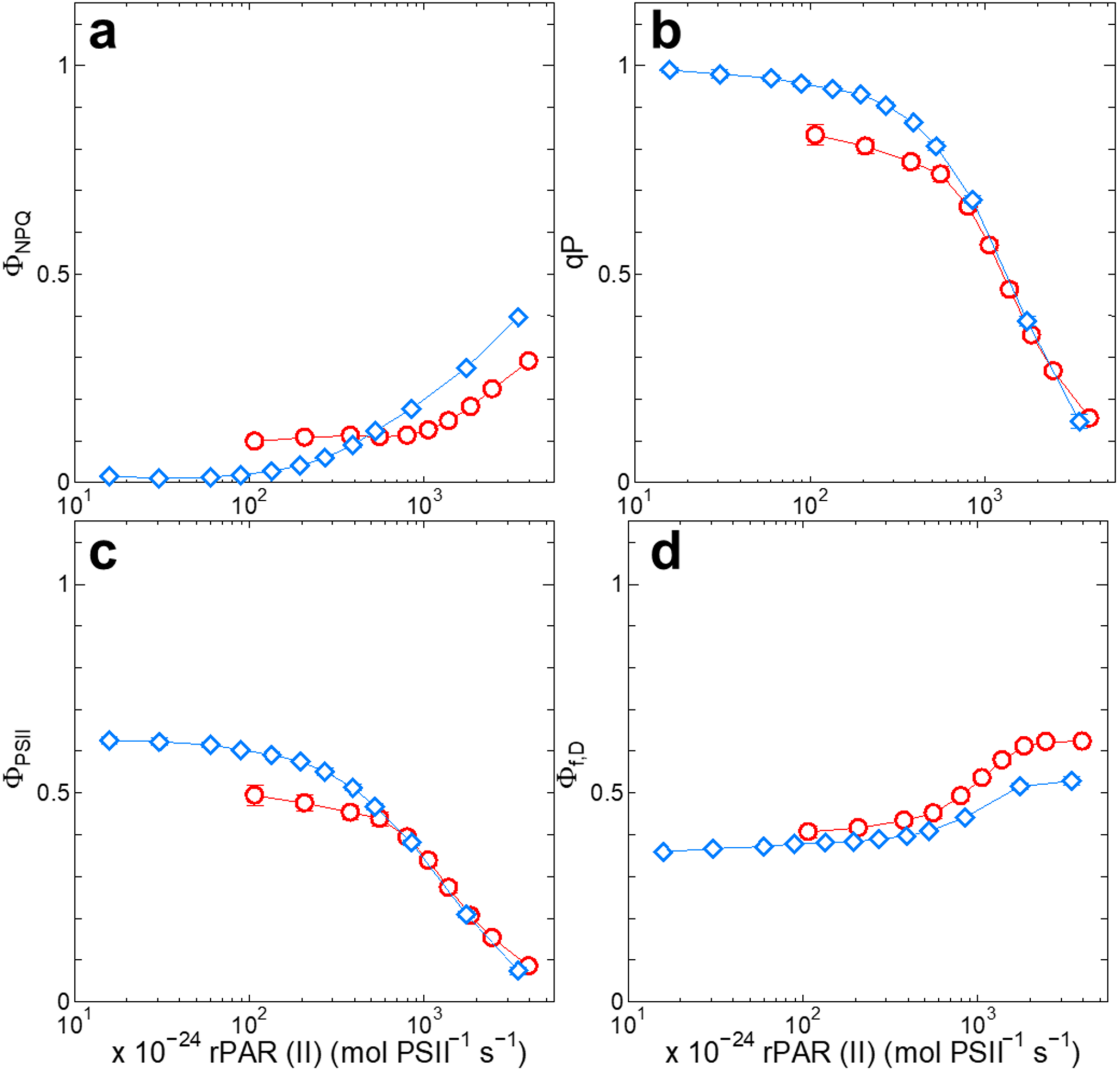
The response of Φ_NPQ_ (**a**), qP (**b**), Φ_PSII_ (**c**) or Φ_f, D_ (**d**) in WT to rPAR (II), the relative rate of photon absorption in PSII core, of blue or orange actinic light. Parameters are plotted on a logarithmic scale. Blue diamonds, measured under blue actinic light; red circles, measured under orange actinic light. Averages ± SD of three independent cultures are presented

To examine the characteristics of the light-dependent induction of Φ_f, D_ under the OCP-activating conditions as well as the OCP-non-activating conditions, we compared the energy partitioning under blue actinic light with that under orange actinic light. Since orange light is much more effective in exciting PSII than blue light in cyanobacteria, we first determined the wavelength-dependent absorption cross-section of PSII, so-called Sigma (II)_λ_. Sigma (II)_625_ was determined under orange actinic light and was more than six times larger than Sigma (II)_440_ under blue actinic light (Table S1), reflecting the small chlorophyll antenna in cyanobacterial PSII. With these Sigma (II)_λ_ values, we corrected the PFD values to the relative one, rPAR (II) (Fig. 3), which is the product of PFD and Sigma (II)_λ_. Under high light with rPAR (II) above approximately 500 × 10^− 24^ mol PSII^− 1^ s^− 1^, Φ_NPQ_ was higher under blue actinic light than under orange actinic light, whereas it was lower under low light (Fig. 3a). It should be noted that state transition, which is regulated by the redox state of the PQ pool (Mullineaux and Allen 1986; Mullineaux et al. 1997), is also reflected in Φ_NPQ_ in cyanobacteria (Campbell and Öquist 1996). Furthermore, the PQ pool in cyanobacteria is reduced in the dark through respiratory electron transport, and Φ_NPQ_ is larger in the dark than in the growth light (Campbell and Öquist 1996). It is plausible to assume that the near-zero Φ_NPQ_ under weak blue actinic light (Fig. 3a) reflected the fully oxidized PQ pool due to preferential excitation of photosystem I (PSI) by weak blue light. The oxidized PQ pool under weak blue actinic light was also suggested by the qP value close to 1 under the same condition (Fig. 3b). By contrast, under high-light at the same rPAR (II), Φ_NPQ_ was higher under blue light compared with that under orange light (Fig. 3a) because of the presence of the OCP-dependent energy dissipation. The difference, however, did not affect the redox state of Q_A_ (Fig. 3b) or the yield of photosynthesis (Fig. 3c) under high-light conditions; the levels of qP and Φ_PSII_ were indistinguishable between blue light and orange light when plotted against rPAR (II). The level of Φ_f, D_ (Fig. 3d) seems to be determined according to the level of the OCP-dependent thermal dissipation, so as to keep the yield of photosynthesis at a certain level irrespective of the actinic light conditions.

## Discussion

In this study, we demonstrated that the absence of the OCP-dependent thermal dissipation did not influence the yield of photosynthesis in cyanobacteria under high-light conditions by the corresponding induction of Φ_f, D_ (Fig. 1a-c). This induction of Φ_f, D_ was not accompanied by the reduction of Q_A_ (Fig. 2b), indicating that the process is not a passive response resulting from the increased fraction of closed PSII reaction centers due to insufficient induction of regulatory non-photochemical quenching as generally believed (Klughammer and Schreiber 2008; Endo et al. 2014). Since the induction of Φ_f, D_ was observed by actinic illumination not only with blue light but also with orange light (Figs. 1f and 3d), the process should be induced to cope with general high-light conditions, and it is not the response to the lack of the OCP-dependent thermal dissipation. Furthermore, after correction with absorption cross-section of PSII, blue and orange actinic light gave identical yield of photosynthesis under high-light conditions (Fig. 3c). It is tempting to assume that the induction of Φ_f, D_ is a regulatory process to keep the yield of photosynthesis at a certain level, irrespective of the induction levels of Φ_NPQ_.

The situation is different in land plants that exhibit constant Φ_f, D_ under steady-state conditions regardless of actinic light PFD (Hendrickson et al. 2004; Ishida et al. 2011; Kono et al. 2014; Barbato et al. 2020). When the zeaxanthin accumulation or the PsbS protein was defective in *A. thaliana* or *O. sativa*, the light-induced increase in Φ_f, D_ was observed (Ishida et al. 2011; Barbato et al. 2020). This increase is, however, accompanied by the reduction of Q_A_, indicating that this response to excess light in land plants is a passive result. We assume that the zeaxanthin- or the PsbS-dependent thermal dissipation at LHCII in land plants is indispensable to avoid the over-reduction of the photosynthetic electron transport chain, and it cannot be replaced by other mechanisms.

The induction of Φ_f, D_ in cyanobacteria can be suppressed by the overexpression of OCP (Fig. 1c) so that the condition comparable to that in land plants can be realized. In such ox-*ocp*, however, the yield of photosynthesis decreased (Fig. 1a). In other words, the yield of photosynthesis can be increased by replacing a part of Φ_NPQ_ with Φ_f, D_ in the case of cyanobacteria. Thus, it would be reasonable to assume that the induction of Φ_f, D_ under high-light conditions is not a mere compensatory response due to the limited cyanobacterial capacity of the OCP-dependent thermal dissipation, but rather a regulated acclimatory process to high-light conditions. At any rate, the complete removal of excess energy via the OCP-dependent mechanism has a drawback in photosynthetic efficiency even under high-light conditions. Apparently, the alternative strategy independent of OCP is employed in cyanobacteria in order to cope with excess light without lowering the yield of photosynthesis. The Φ_f, D_-reflecting energy dissipation should be a mechanism at the core level rather than at the antenna level, since the light-induced increase in Φ_f, D_ reflected the increase in chlorophyll fluorescence rather than phycobilisome fluorescence (Fig. S4). More detailed mechanisms of the increase in Φ_f, D_ are unknown. It was reported that the yield of fluorescence from cyanobacterial PSII core complexes increased by some structural changes upon actinic illumination without Q_A_ reduction (Sipka et al. 2021). There may be some common points between the two phenomena, but the comparison seems to be a rather far shot. At all events, the fluorescence emission itself cannot act as a mechanism of energy dissipation considering the low yield of chlorophyll fluorescence.

If the increase in Φ_f, D_ can completely dissipate the excess energy, the OCP-dependent thermal dissipation at the antenna level may be unnecessary. Photosynthetic performance seems to be maintained without OCP under growth light or short-term (within minutes) high-light conditions, since Δ*ocp* exhibits similar or slightly higher Φ_PSII_ compared to WT regardless of actinic PFD (Fig. 1a). This is consistent with the previous reports that the rate of whole-chain electron transport from PSII to PSI in Δ*ocp* of *Synechocystis* 6803 is similar to that in WT regardless of actinic PFD (Kusama et al. 2015; Takahashi et al. 2019). When high-light exposure is prolonged (> 10 min), however, loss in the PSII activity is more pronounced in Δ*ocp* of *Synechocystis* 6803 than in WT (Wilson et al. 2006; Kusama et al. 2015). Furthermore, cyanobacterial species originally lacking *ocp* genes, such as *Synechococcus elongatus* PCC 7942 and *Thermosynechococcus elongatus* BP-1, are more sensitive to long-term exposure to high light than *Synechocystis* 6803 having OCP (Boulay et al. 2008). These observations indicate that the OCP-dependent thermal dissipation is required for the photoprotection of PSII under long-term (> 10 min) high light in cyanobacteria, while it can be replaced by the Φ_f, D_-related mechanism under short-term (within minutes) high light.

It must be noted that the basal expression level of OCP in WT of *Synechocystis* 6803 does not appear to be high enough to achieve the maximum photoprotective potential, judging from the earlier report that the overexpression of OCP further alleviated the photoinhibition of PSII upon exposure of *Synechocystis* 6803 to high light at 1500 µmol m^− 2^ s^− 1^ for more than 30 min (Takahashi et al. 2019). However, such high-light conditions are not so frequently experienced in aquatic environments of cyanobacteria. We assume that the priority is given to preventing the decrease in efficiency of photosynthesis under frequent short-term high light than to avoiding photoinhibition under rare persistent high light in *Synechocystis* 6803.

In addition to cyanobacteria, several eukaryotic algae including green algae such as *Chlamydomonas reinhardtii* (Ait Ali et al. 2008; Fu et al. 2023) and *Chlorella sorokiniana* (Schuurmans et al. 2015), or glaucophyte *Cyanophora paradoxa* (Misumi and Sonoike 2017) exhibit the light-dependent increase in the steady-state level of Φ_f, D_ or Fs. In the model green algae *C. reinhardtii*, overexpression of the PsbS and Lhcsr proteins, which are involved in the induction of the thermal dissipation at the antenna level, lowered not only Φ_f, D_ but also the efficiency of photosynthesis under high light (Wilson et al. 2023) as observed in ox-*ocp* of *Synechocystis* 6803 (Fig. 1). This suggests that the over-removal of light energy at the antenna level may cause loss rather than gain of the photosynthetic performance even under high-light conditions in *C. reinhardtii*. This is in contrast to the case of land plants, such as *A. thaliana* or *Chrysanthemum morifolium*, in which an enhancement of the zeaxanthin-dependent thermal dissipation did not lower but elevated the yield of photosynthesis (Han et al. 2023, 2024). Based on these observations, loss of the photosynthetic performance resulting from over-induction of the thermal dissipation at the antenna level seems to be specific to the photosynthetic organisms with the light-induced increase in the steady-state level of Φ_f, D_ such as cyanobacteria and green algae.

In cyanobacteria as well as green algae and glaucophyte, the state transition is a critical acclimatory process with large capacity and have much higher contribution to the parameters representing regulatory non-photochemical quenching, when comparing to land plants (Schreiber et al. 1995; Campbell and Öquist 1996; Misumi and Sonoike 2017). Thus, as strategies to relieve the over-reduction of photosynthetic electron transport chain under high-light conditions, these organisms may prefer the allocation of energy between photosystems through state transition rather than the removal of energy at the antenna level, i.e., OCP-dependent thermal dissipation, in the case of cyanobacteria. Further studies are necessary to reveal the possible involvement of state transition in the regulation of Φ_f, D_ in addition to the OCP-dependent thermal dissipation investigated in this study. Compared to land plants, cyanobacteria and the eukaryotic algae have abundant alternative electron sinks, such as flavodiiron proteins (Allahverdiyeva et al. 2013, 2015; Ilík et al. 2017), the respiratory electron transport chain (Howitt and Vermaas 1998; Berry et al. 2002; Lea-Smith et al. 2013), or chlororespiration (Cournac et al. 2002; Misumi and Sonoike 2017). These alternative electron sinks may be the reason why the removal of excess energy at the antenna level can be partially compensated by the Φ_f, D_-related mechanism in these photosynthetic organisms.

## Electronic supplementary material

Below is the link to the electronic supplementary material.

Supplementary Material 1

## Data Availability

Data is provided within the manuscript or supplementary information files.
