# Supplementary material for: Light-induced increase in the steady-state chlorophyll fluorescence in cyanobacteria reflects induction of energy dissipation complementary to orange carotenoid protein-dependent thermal dissipation
Source: Photosynth Res. 2025 Jul 9;163(4):38. doi: 10.1007/s11120-025-01159-0 (PMC12241190; doi:10.1007/s11120-025-01159-0)
Supplement: Supplementary file 1 — Supplementary Material 1 [file 11120_2025_1159_MOESM1_ESM.pdf]

Supplementary Information

Light-induced increase in the steady-state chlorophyll fluorescence in cyanobacteria reflects induction of energy dissipation complementary to orange carotenoid protein-dependent thermal dissipation

Takako Ogawa[1, 2], Hiroko Takahashi[1], Yoshitaka Nishiyama[1], Yukako Hihara[1], Kintake Sonoike[2, *]

[1] Graduate School of Science and Engineering, Saitama University, 255 Shimo-Okubo, Sakura-ku, Saitama 338-8570, Japan

[2] Faculty of Education and Integrated Arts and Sciences, Waseda University, 2-2 Wakamatsu-cho, Shinjuku-ku, Tokyo 162-8480, Japan

[*]Corresponding author: E-mail, sonoike@waseda.jp

Figure S1

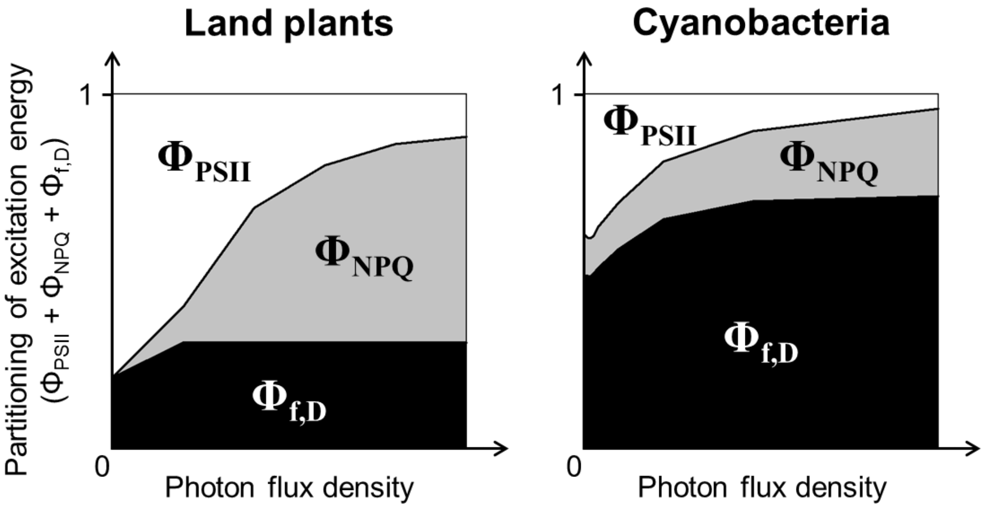

Fig. S1 Schematic model of the typical partitioning of excitation energy to photosynthesis (Φ_{PSII}; white), the regulated thermal dissipation (Φ_{NPQ}; gray) or non-regulated energy dissipation ($\Phi_{f,D}$; black) in land plants (left panel) and cyanobacteria (right panel). Because the model of cyanobacteria is based on the energy partitioning under physiological condition (i.e., under white/orange light, not blue light), there is a contribution of phycobilisome fluorescence to the fluorescence signal

Figure S2

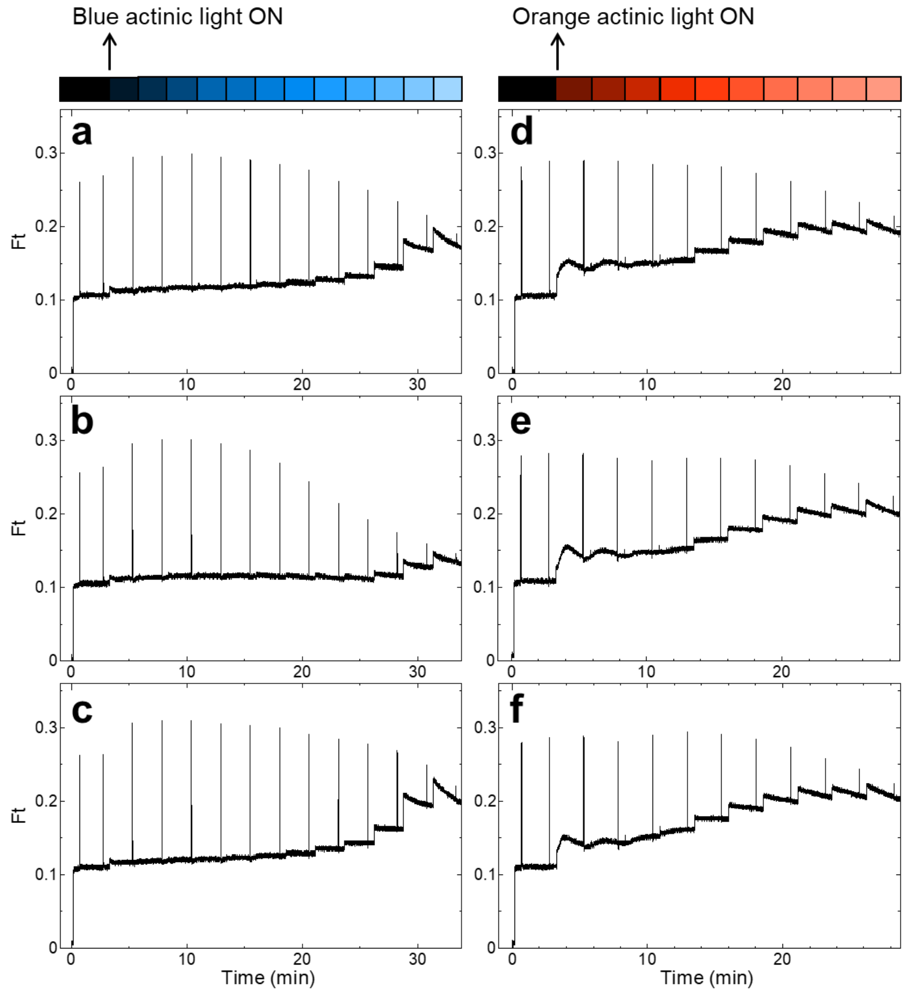

Fig. S2 Trace of time-dependent fluorescence level (Ft) for the measurements of Fs and Fm' under blue (a-c) or orange (d-f) actinic light in WT (a, d), ox-*ocp* (b, e) and Δ*ocp* (c, f). Cell suspensions were exposed to blue or orange actinic light with increasing PFD ranging from 14 to 3055 μmol m^{-2} s^{-1} or from 15 to 554 μmol m^{-2} s^{-1}, respectively. Colors of the bars on the top of the figures indicate illumination condition; dark (black), illumination with blue actinic light (blue) or illumination with orange actinic light (orange). Fluorescence signal was detected by the measuring light at 440 nm

Figure S3

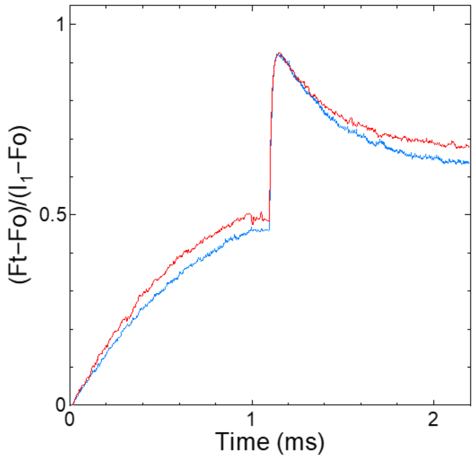

Fig. S3 Increase kinetics of fluorescence yield upon illumination with orange actinic light (510 µmol m^{-2} s^{-1}; red line) or blue actinic light (3123 µmol m^{-2} s^{-1}; blue line) in the presence of background far red light. Single-turnover pulse for 50 µs was applied 1 ms after the onset of actinic light to determine the maximum fluorescence level (I_1-level) that can be reached in the presence of an oxidized PQ pool. Time-dependent fluorescence levels (Ft) were normalized by Fo-level and I_1-level as 0 and 1, respectively. The averaged kinetics of three independent cultures are presented

Figure S4

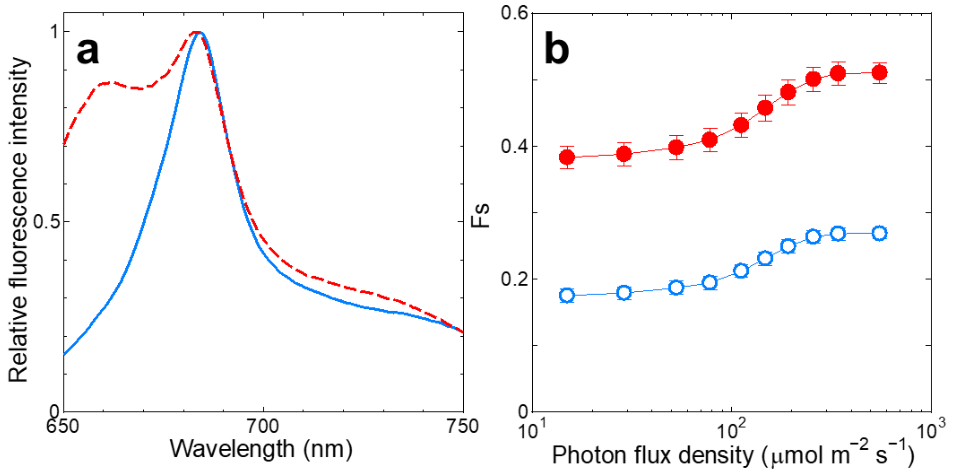

Fig. S4 Fluorescence emission spectra of cyanobacterial cells measured at room temperature (a) and the response of Fs to PFD of orange actinic light (b). (a) Each spectrum was normalized at the chlorophyll fluorescence peak height around 685 nm. Red dashed line, phycocyanin excitation at 625 nm; blue solid line, chlorophyll excitation at 440 nm. The averaged spectra of three independent cultures are presented. (b) Fs is plotted against logarithmic scale. Open blue circles, detected by the measuring light at 440 nm; closed red circles, detected by the measuring light at 625 nm. Averages ± SD of three independent cultures are presented

Table S1. Tau and Sigma(II)λ estimated by rising kinetics of fluorescence upon illumination with actinic light

**Wavelength (nm)**	**PFD (μmol m^{-2} s^{-1})***	***Tau* (ms)**	**Sigma (II)λ (nm^2 PSII^{-1})**
440	3123	0.470 ± 0.032	1.13 ± 0.08
625	510	0.458 ± 0.046	7.16 ± 0.71

* To minimize potential errors in the fitting process due to the difference in the rate of fluorescence induction, Sigma (II)λ was calculated with Tau determined under 510 μmol m^{-2} s^{-1} in the case of orange actinic light and 3123 μmol m^{-2} s^{-1} in the case of blue actinic light, which gave a similar rate of fluorescence induction (Fig. S3).
